# Risk Factors Associated with the Occurrence of Autoimmune Diseases in Adult Coeliac Patients

**DOI:** 10.1155/2018/3049286

**Published:** 2018-09-12

**Authors:** Laura Conti, Edith Lahner, Gloria Galli, Gianluca Esposito, Marilia Carabotti, Bruno Annibale

**Affiliations:** Medical-Surgical Department of Clinical Sciences and Translational Medicine, Sapienza University of Rome, Rome, Italy

## Abstract

**Objectives:**

Autoimmune diseases (AD) may be associated with coeliac disease (CD), but specific risk factors have been poorly investigated. The aim of this study was to assess the spectrum of AD and its specific risk factors associated in a series of adult coeliac patients.

**Materials and Methods:**

We performed a single-center case-control study including adult newly diagnosed CD patients. To evaluate the risk factors of the association between AD and CD, 341 coeliac patients included were categorized on the basis of AD presence: 91 cases with at least one AD and 250 controls without AD were compared for clinical, serological, and histological features. Eighty-seven cases were age-gender-matched with 87 controls.

**Results:**

Among 341 CD patients, 26.6% of CD patients had at least one AD. Endocrine and dermatological diseases were the most prevalent AD encountered: autoimmune thyroiditis was present in 48.4% of cases, psoriasis in 17.6%, and type I diabetes and dermatitis herpetiformis in 11%, respectively. At logistic regression, factors associated with AD were a positive 1st-degree family history of AD (OR 3.7, 95% CI 1.93–7), a body mass index ≥ 25 kg/m^2^ at CD diagnosis (OR 2.95%, CI 1.1–3.8), and long standing presentation signs/symptoms before CD diagnosis (>10 years) (OR 2.1, 95% CI 1.1–3.7). Analysis on age-gender-matched patients confirmed these results.

**Conclusions:**

CD patients with family history of AD, overweight at CD diagnosis, and a delay of CD diagnosis had an increased risk of having another AD. The benefit of CD screening in these specific subsets of patients with AD awaits further investigation.

## 1. Introduction

Coeliac disease (CD) is an immune-mediated enteropathy triggered by the ingestion of gluten-containing grains in genetically susceptible persons. CD is one of the most common causes of chronic malabsorption and affects approximately 1% of the Western population [1, 2]. In CD patients, a significantly increased prevalence of autoimmune disorders (AD), including systemic diseases and organ-specific diseases, has been reported compared to healthy controls, with an estimated prevalence ranging from 15 to 30% [3, 4]. Conversely, a significantly increased prevalence of CD has been documented in individuals with several AD. Screening for CD is recommended in patients affected by type I diabetes mellitus and autoimmune thyroid disease [5]. The association among CD and AD has been suggested to be due to shared genetic features between CD and other immune-mediated conditions, similar environmental triggers, and the loss of intestinal barrier [6, 7]. Previous studies have examined factors associated with the coexistence of AD among CD patients providing conflicting results [8–10]. The aim of the present study was to assess the spectrum of AD and its specific risk factors associated in a series of adult CD patients.

## 2. Materials and Methods

### 2.1. Study Population and Design

**Figure 1:**
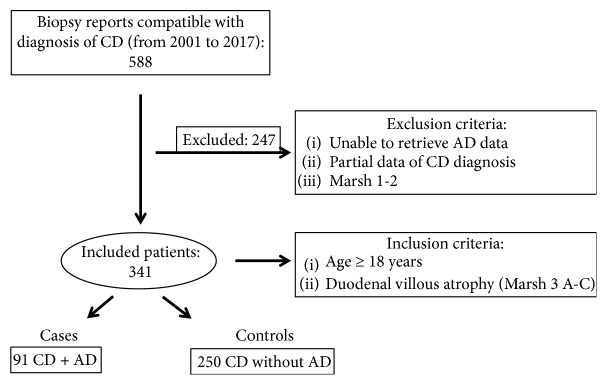
Flowchart selection of study population. Finally, 341 coeliac patients meeting the inclusion criteria were categorized into two groups: 91 cases (coeliac patients with autoimmune diseases) and 250 controls (coeliac patients without autoimmune diseases). CD: coeliac disease; AD: autoimmune disease.

This was a single-center case-control study reported according to the checklist items indicated in the STROBE guidelines [11]. Records of patients who consecutively underwent oesophago-gastro-duodenoscopy with duodenal biopsies and had histological features compatible with CD at the referral Coeliac Disease Center of Sant'Andrea University Hospital, Rome, from 2001 to 2017 were assessed for eligibility. The study was database driven, and data from 341 CD patients (median age 35, range 18–76 years, females, 77.4%) with the following inclusion criteria were considered: CD diagnosis in adulthood (>18 years at time of gastroscopy) with duodenal villous atrophy (Marsh 3 A-C) with complete clinical and biochemical data recorded at the first visit. Patients with incomplete serological or histological data for CD diagnosis, partial clinical and personal data, and patients with histological reports compatible with nonatrophic CD (Marsh 1-2) were excluded (Figure 1). At the time of CD diagnosis, a structured questionnaire comprising personal and clinical data including associated AD was filled in for each patient.  Table 1 showed baseline characteristics of included patients. The diagnosis of AD (autoimmune thyroiditis, type 1 diabetes mellitus, dermatological diseases such as dermatitis herpetiformis, psoriasis, alopecia, and vitiligo, autoimmune liver diseases, pernicious anemia, systemic lupus erythematosus, rheumatoid arthritis, inflammatory bowel diseases, multiple sclerosis, and others) was based on the evaluation of the clinical history and the revision of clinical charts and/or reports from previous specialist examinations. To evaluate the risk factors of the association between AD and CD, all included patients were categorized on the basis of the presence or absence of AD.

As shown in the flowchart (Figure 1), of the 341 CD patients included, 91 patients (cases) with at least one AD (26.6%) and 250 CD patients without AD (controls) were evaluated.

The diagnosis of CD was based on small intestinal biopsies (*n* = 5) taken from the bulb and the second part of the duodenum and CD specific serological assays. Duodenal histological damage was classified according to Oberhuber et al. and Marsh [12, 13], and only patients with duodenal villous atrophy (Marsh 3 A-C) were included.

According to Oslo classification, CD presentation was classified as (1) classical CD with malabsorption syndrome (diarrhea, weight loss); (2) nonclassical CD with gastrointestinal symptoms (except for diarrhea) and extraintestinal manifestations; and (3) subclinical CD with signs and symptoms below the threshold of detection [14]. At the first visit, serological and histological data were recorded, weight and height for the calculation of body mass index (BMI) were determined in each patient and classified; according to WHO, normal weight if BMI was >18.5 kg/m^2^ to ≤24.9 kg/m^2^, underweight if was BMI< 18.5 kg/m^2^, while overweight if BMI was ≥25 kg/m^2^.

In addition, all patients underwent serological studies including CD specific serological assays with anti-transglutaminase antibodies (tTG-IgA), anti-endomysial antibodies (EMA-IgA) together with anti-transglutaminase antibodies (Ttg-IgG) in case of IgA deficiency. Genetic testing was obtained in selective cases of incongruity between histological and serological features. All patients were tested for the following laboratory indices: haemoglobin, albumin, cholesterol, triglycerides, and ferritin (anemia was defined as haemoglobin < 12 g/100 ml in women and <13.5 g/100 ml in men). Thyroid hormones (triiodothyronine, free thyroxin, and basal thyrotropin) and antiperoxidase antibodies were routinely assessed in each patient at CD diagnosis time, and the diagnosis of autoimmune thyroiditis was made as previously reported [15, 16].

#### 2.1.1. Statistical Analyses

For the computation of sampling, data from the previously published papers of AD prevalence in coeliac patients were used [4, 17]. Assuming a normal distribution, we calculated that 343 patients would have been required to yield a statistical power of 95% and a probability of type I error of 0.05 and type II error of 0.01. Data were expressed as medians, ranges, and percentages. Comparisons between cases and controls were made using Fisher's exact test, chi-square test, and Mann–Whitney *U* test, as appropriate. At univariate analysis, the two groups were compared for clinical features including gender, age at CD diagnosis, BMI, signs and symptoms leading to CD diagnosis and their duration before CD diagnosis (more than 10 years), 1st-degree family history for CD or AD, and presence of severe duodenal atrophy (March 3C) as well as serological features including haemoglobin, albumin, cholesterol, triglycerides, and ferritin. Odds ratios (ORs) and 95% confidence intervals (CIs) were used to describe the associations and were obtained by logistic regression analysis performed on the whole group of patients. Two-tailed *p* values < 0.05 were considered statistically significant. According to the STROBE guidelines, to increase the study's efficiency by ensuring similarity in the distribution of variables between cases and controls [11], we repeated a logistic regression analyses by using the above cited covariants on controls individually matched to cases by gender and age (years ±1) at CD diagnosis. For 87 cases, one control was identified who met all matching criteria (1 : 1). When more than one control was available, we used a random selection method considering the lower number of the histology report. Four cases were excluded because they did not have adjusted controls meeting matching criteria. Statistical analyses were performed using a dedicated Software (MedCalc software, Mariakerke, Belgium, version 12.7.8).

## 3. Results

### 3.1. AD Associated with CD

Overall, in 26.6% of included CD patients at least one AD was associated. The most prevalent AD were endocrine and dermatological AD. As shown in detail in Table 2, autoimmune thyroiditis was present in 48.4% of cases, while psoriasis in 17.6% and type I diabetes and dermatitis herpetiformis in 11%, respectively. Only one AD was present in 81.3% (*n* = 74), and more than one AD in 18.7% (*n* = 17) of cases; when more than one AD was associated, autoimmune thyroiditis was the most frequent (64.7%). In 82 (90%) of the cases, the diagnosis of AD was already known at the CD diagnosis time, whereas in nine patients, the diagnosis of AD was performed concomitantly as part of the diagnostic work-up for CD (within 3 months from CD diagnosis).

### 3.2. Risk Factors for the Concomitant Presence of AD in CD Patients

Comparing cases and controls, CD patients with AD presented more frequently with overweight at CD diagnosis time (29.8% versus 17.2%; *p* = 0.019), with signs/symptoms leading to CD diagnosis persisting for more than 10 years before CD diagnosis (39% versus 26.3%; *p* = 0.03) and with a positive 1st-degree family history for AD (33.7% versus 13.2%; *p* < 0.0001). No differences were found among cases and controls with regard to gender, median age at CD diagnosis, 1st-degree family history of CD, severity of duodenal histological damage (Marsh 3C), and presence of gastrointestinal symptoms at CD diagnosis.

Moreover, among cases and controls, the proportions of nonclassical, classical, and subclinical subtypes according to the Oslo classification were similar with the following percentages for cases and controls, respectively: nonclassical CD (61.5% versus 53.6%; *p* = 0.21); classical CD (28.6% versus 35.6%; *p* = 0.24), and subclinical CD (12% versus 12%; *p* = 1). Concerning nutritional parameters, univariate analysis showed that between the two groups, there were no significant differences of albumin, cholesterol, triglyceride, ferritin, and haemoglobin values.

At logistic regression model, factors linked to the association of AD and CD were a positive 1st-degree family history of AD (OR 3.7, 95% CI 1.93 to 7), a body mass index ≥ 25 kg/m^2^ (OR 2, 95% CI 1.1 to 3.8), and long standing presentation signs/symptoms before CD diagnosis (>10 years) (OR 2.1, 95% CI 1.1 to 3.7), while other features as gender, age ≥ 40 years, clinical presentation, or presence of severe duodenal atrophy (March 3C) were not associated. The logistic regression model performed on age-gender-matched cases and controls confirmed the same risk factors found above (Table 3).

## 4. Discussion

In this case-control study, nearly one-third of our series of adult coeliac patients (26.6%) had at least one cooccurring AD, confirming the previously reported data [10]. A genetic overlap between CD and other immune-mediated conditions could be a plausible explanation for this increased prevalence in coeliac patients compared to healthy controls [6, 7]. Genome-wide association studies (GWAS) have, indeed, suggested common genetic bases located in coding and noncoding DNA regions for CD and other AD such as type I diabetes, ulcerative colitis, rheumatoid arthritis, and Crohn's disease [18, 19]. Endocrine and dermatological AD were the most common associated AD in our series, occurring in 15.8% and 11.7%, respectively, of all included patients. More specifically, autoimmune thyroiditis (12.8%), psoriasis (4.6%), and type I diabetes (2.9%) were the most frequent AD keeping in step with the previously reported data [3, 20]. The prevalence of dermatitis herpetiformis was instead lower (2.9%) than that observed in most previous studies (5–10%); this may possibly be explained by the decreasing occurrence of dermatitis herpetiformis as found by an Italian larger series (4%) [21] and in a recent Dutch study (3.2%) [4].

We found the following risk factors linked to the cooccurrence of AD in coeliac patients: family history of autoimmunity, overweight at CD diagnosis, and a delayed diagnosis of CD with a long interval between the first signs/symptoms leading to CD diagnosis and CD diagnosis. With regard to family history of AD, we found a presence of autoimmune diseases of 33.7% among first-degree family members of cases and 13.2% of first-degree family members of controls, percentages that in both cases resulted higher than the general population prevalence all over the world (3–5%). A previous paper showed a 2.4-fold risk (CI 95% 1.71–3.31) to develop AD in pediatric and adult CD patients with positive family history [8]. Indeed, among several risk factors associated with the onset of AD, family history of AD, also known as familial autoimmunity, has been documented showing how AD cluster in families [22]. In fact, one study [23] observed a significantly increased risk of noncoeliac AD in first-degree relatives of coeliac patient explaining this phenomenon by genetic factors but also by ascertainment bias and environmental factors.

In our series, coeliac patients with an associated AD presented more frequent overweight at CD diagnosis time (31%) than patients with CD alone (17.2%). To the best of our knowledge, only one cross-sectional study previously investigated the relation between BMI and autoimmune disorders in CD, in particular with immune-mediated skin diseases, demonstrating that overweight was positively related to the prevalence of dermatitis herpetiformis and psoriasis in adults with untreated CD [24]. Taking into consideration only the overweight cases of the present study, we found that autoimmune thyroiditis was the most prevalent AD in accordance with the above results (16/28) and only three and four patients presented with psoriasis and dermatitis herpetiformis, respectively. Eventual confounding factors possibly explaining overweight were found in five of 28 overweight cases: two patients had a diagnosis of type I diabetes in childhood and were insulin dependent and three patients had a diagnosis of autoimmune thyroiditis during CD diagnosis work-up and they still were not on levothyroxine treatment at the first CD visit time, perhaps explaining their weight increase due to an initial hypothyroidism. In addition, nutritional parameters were similar between overweight and normal weight patients. We are not able to explain this association but we can speculate that the proinflammatory condition present in overweight may favor the development of several diseases, including also AD, due to the key role of white adipose tissue as an active endocrine organ, playing a role in immune and inflammatory processes by releasing proinflammatory mediators in common with immune-mediated diseases [25].

Another observed risk factor was the delay of CD diagnosis after onset of signs/symptoms among cases. In literature, there are conflicting reports on the relation between a longer exposure to gluten in patients with higher age at CD diagnosis and the increased risk to develop AD. Previous Italian [9] and French [8] studies showed a role of these two factors, not confirmed by other two studies from Italy [10] and Finland [26].

In our study, no difference was found among cases and controls with regard to median age at CD diagnosis even if CD patients with associated AD had a delayed diagnosis of CD with a long interval between the first signs/symptoms leading to CD diagnosis and CD diagnosis.

Albeit, it is not clear whether coexistence of AD in coeliac patients could be considered a comorbidity with their own specific de novo dysreactivity of the immune system rather than CD-dependent diseases as a consequence of inflammation of the gut induced by gluten. Taking together these data, the duration of untreated disease might be the real risk factor rather than the age of patients at the time of CD diagnosis. In turn, this might be due to a decreasing number of patients with classical CD (diarrhea, weight loss) [21] as also supported by our study, in which the nonclassical CD was the most frequent presentation form. This changing clinical profile and the underestimation of nonclassical gastrointestinal symptoms, often attributed to the already known AD, might be one possible explanation for a delayed diagnosis of CD.

We are aware of some limitations of this study. First, although coeliac patients were included prospectively, this study was mainly database driven and data were analyzed retrospectively. The aim of our study was to analyze factors associated with an increased risk of AD among adult coeliac patients; however, in this case-control study, the lack of a healthy control group did not allow to evaluate the relative risk of this association.

## 5. Conclusion

In summary, the present study confirms the wide spectrum of different AD among CD patients and shows that the risk factors for associated AD were positive family history of AD, overweight at CD diagnosis time, and a delay of CD diagnosis. The benefit of CD screening in these specific subsets of patients with AD awaits investigation.

## Figures and Tables

**Table 1:** Baseline characteristics of 341 included coeliac patients.

Female gender	77.4
Age at coeliac disease diagnosis, years, median (range)	35 (18–76)
Body mass index, kg/m^2^, median (range)	21.3 (14.5–38.2)
Gastrointestinal symptoms leading to coeliac disease diagnosis	70.7
(i) Classical presentation	33.7
(ii) Nonclassical presentation	55.7
(iii) Subclinical presentation	12
Marsh 3C	52.8
Family history of coeliac disease	16.3
Family history of autoimmune disease	18.7

Data are expressed as percentage where not otherwise indicated.

**Table 2:** Spectrum of autoimmune diseases observed in 91 adult patients with coeliac disease.

	Number and percentages (%) of AD in 91 cases	Percentages of AD in all included CD patients
Endocrine diseases	54 (59.3)	15.8
Autoimmune thyroid diseases	44 (48.4)	12.9
Type I diabetes mellitus	10 (11)	2.9

Dermatological diseases	40 (44)	11.7
Psoriasis	17 (18.7)	4.9
Dermatitis herpetiformis	10 (11)	2.9
Alopecia areata	7 (7.7)	2
Vitiligo	4 (4.4)	1.2
Lichen sclerosus	2 (2.2)	0.6

GI diseases	3 (3.3)	0.9
Autoimmune hepatitis	1 (1.1)	0.3
Inflammatory bowel diseases	1 (1.1)	0.3
Pernicious anemia	1 (1.1)	0.3

Rheumatological/connective tissue diseases	4 (4.4)	1.2
Rheumatoid arthritis	1 (1.1)	0.3
Spondyloarthritis	1 (1.1)	0.3
Systemic lupus erythematosus	1 (1.1)	0.3
Connettivitis	1 (1.1)	0.3

Others	2 (2.2)	0.6
Sarcoidosis	1 (1.1)	0.3
Multiple sclerosis	1 (1.1)	0.3

AD: autoimmune disease; CD: coeliac disease.

**Table 3:** Risk factors for the association of autoimmune diseases (AD) in coeliac disease (CD) patients by logistic regression analysis in the whole group of included patients and in gender-age-matched patients.

	All included patients (*n* = 341) OR (95% CI)	Gender-age matched patients (*n* = 172) OR (95% CI)
*Age at CD diagnosis*		
<40	1.0	—
≥40	1.2 (0.7–2.1)	
*Gender*		
Male	1.0	—
Female	0.65 (0.3–1.3)	
*Overweight (BMI ≥ 25 kg/m^2^)*		
No	1.0	
Yes	2 (1.1–3.8)	8.4 (2.9–24.8)
*Gastrointestinal symptoms leading to CD diagnosis*		
No	1.0	
Yes	1.2 (0.6–2.3)	0.6 (0.2–1.6)
*Severe histological damage (Marsh 3C)*		
No	1.0	
Yes	0.95 (0.6–1.7)	0.96 (0.4–2.15)
*Family history of AD*		
No	1.0	
Yes	3.7 (1.9–7)	6.9 (2.2–21.7)
*Duration of symptoms ≥ 10 years before CD diagnosis*		
No	1.0	
Yes	2.1 (1.1–3.7)	19.6 (5.3–73.1)

## Data Availability

The data used to support the findings of this study are included within the article.
